# Lack of recognition of genetic biodiversity: International policy and its implementation in Baltic Sea marine protected areas

**DOI:** 10.1007/s13280-016-0776-7

**Published:** 2016-04-20

**Authors:** Linda Laikre, Carina Lundmark, Eeva Jansson, Lovisa Wennerström, Mari Edman, Annica Sandström

**Affiliations:** 1 Department of Zoology, Division of Population Genetics, Stockholm University, 106 91 Stockholm, Sweden; 2 Department of Business Administration, Technology and Social Sciences, Luleå University of Technology, 971 87 Luleå, Sweden; 3 Institute of Marine Research, 5817 Bergen, Norway

**Keywords:** Conservation policy implementation, Convention on Biological Diversity, CBD, Helsinki Convention, Genetic variation, MPA

## Abstract

Genetic diversity is needed for species’ adaptation to changing selective pressures and is particularly important in regions with rapid environmental change such as the Baltic Sea. Conservation measures should consider maintaining large gene pools to maximize species’ adaptive potential for long-term survival. In this study, we explored concerns regarding genetic variation in international and national policies that governs biodiversity and evaluated if and how such policy is put into practice in management plans governing Baltic Sea Marine Protected Areas (MPAs) in Sweden, Finland, Estonia, and Germany. We performed qualitative and quantitative textual analysis of 240 documents and found that agreed international and national policies on genetic biodiversity are not reflected in management plans for Baltic Sea MPAs. Management plans in all countries are largely void of goals and strategies for genetic biodiversity, which can partly be explained by a general lack of conservation genetics in policies directed toward aquatic environments.

**Electronic supplementary material:**

The online version of this article (doi:10.1007/s13280-016-0776-7) contains supplementary material, which is available to authorized users.

## Introduction

Genetic diversity is the foundation for all biological diversity; the persistence and evolutionary potential of species rely on it for adaptation to natural and human-induced selective pressures (Allendorf et al. 2012). Conservation genetics research indicates links between variation at the DNA level (genetic variation) of species and biological productivity and diversity (Reusch et al. 2005), resilience to environmental stressors (Frankham 2005; Hellmair and Kinziger 2014), and adaptation to changing environmental features including climate change (McGinnity et al. 2009; Barshis et al. 2013). In some systems, intraspecific variation (i.e., genetic variation within and between populations of a species) provides similar biological function as species diversity (Cook-Patton et al. 2011). This knowledge is of key importance for sustainable management and is recognized in the Convention on Biological Diversity (CBD; www.cbd.int).

Studies indicate that CBD implementation concerning genetic diversity lags behind implementation for other levels of biodiversity (Laikre et al. 2010). Similarly, scientific knowledge on genetic biodiversity is often not used in practical management of biological resources in spite of being of direct relevance for reaching management goals (Sandström 2010, 2011; Sevä 2013), indicating that management is not adaptive with respect to genetic diversity. *Adaptive management* is a guiding principle in contemporary environmental policy and resource management, implying a close link between science, policy, and management; the management consciously learns and *adapts* to new knowledge to reduce uncertainty and attain more robust decision-making processes (Holling 1978; Folke et al. 2002).

The Baltic Sea represents a system where genetic diversity is expected to be of particular concern (Johannesson et al. 2011). It is evolutionary young, formed less than 10 000 years ago (Zillén et al. 2008), with brackish water to which relatively few marine and freshwater species have adapted. In its species-poor environment, important ecosystem functions are upheld by single or a few species (Elmgren and Hill 1997), and genetic diversity within species constitutes a potentially more important part of biodiversity as compared to high species diversity systems (Laikre et al. 2008).

Relatively extensive knowledge on genetic diversity is available for several Baltic Sea species. Studies have shown that adaptation to Baltic Sea conditions appears to have resulted in (i) genetically unique make-ups implying that Baltic populations are typically genetically distinct from populations of the same species outside of the Baltic (Johannesson and André 2006), (ii) lower genetic variation than populations in the Atlantic Ocean (Johannesson et al. 2011), and (iii) species-specific patterns of genetic variation within the Baltic apparently reflecting a variety of evolutionary histories and patterns of genetic drift and gene flow (Laikre et al. 2005; Wennerström et al. 2013). These characteristics in combination with low species diversity make Baltic Sea biodiversity particularly sensitive to anthropogenic stressors.

Human-induced pressures are extensive in the Baltic and include high levels of nutrients, oil, heavy metals, and toxins (Jansson and Dahlberg 1999; Lehtonen and Schiedek 2006; Ducrotoy and Elliott 2008), habitat modification, and fragmentation including large areas of oxygen-depleted sea beds, large-scale fishing and stocking (Diaz and Rosenberg 2008; Palmé et al. 2012), spread of alien species (Björklund and Almqvist 2010), and climate change effects on salinity and water temperature (Meier 2006; Neumann 2010). These pressures are expected to increase the importance of genetic variation as a basis for population and species adaptation and resilience (Johannesson et al. 2011).

In this paper, we investigate *if* and *how* genetic biodiversity is taken into consideration in implementing international conservation policy in national and regional Baltic Sea management. The Baltic Sea shore encompasses 9 countries: Sweden, Finland, Russia, Estonia, Latvia, Lithuania, Poland, Germany, and Denmark. All of them are parties to the CBD and to the Convention on the Protection of the Marine Environment of the Baltic Sea Area (the Helsinki Convention), and all except Russia are part of the European Union (EU) which has its own environmental legislation including the Habitats Directive that is aimed at protecting threatened habitats and species (Directive 92/43/EEC). Implementation of the common international policy framework is thus incorporated into many different national contexts. In this study, we focus on national implementation in Sweden, Finland, Estonia, and Germany.

With respect to regional management, we focus on marine protected areas (MPAs) because they constitute an important tool for biodiversity conservation in the marine environment (Semmens et al. 2010). Our study includes (i) documenting the extent of genetic considerations including how concerns regarding genetic variation are formulated in international policies that govern the Baltic Sea and its biodiversity, (ii) investigating if and how international policies are transformed into national policy in the four countries, and (iii) evaluating if and how international and national policies regarding gene level biodiversity are transformed into management plans governing Baltic Sea MPAs in the four countries.

## Materials and methods

**Fig. 1:**
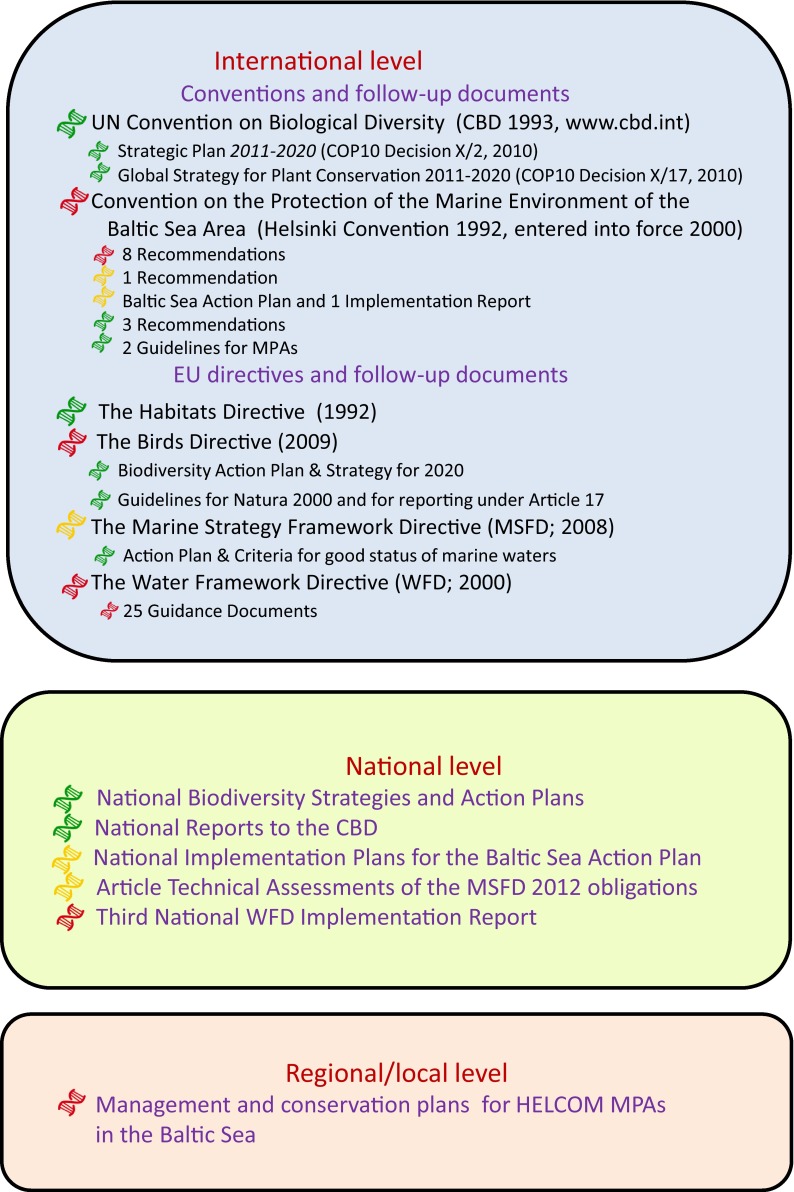
Documents at the international, national, and regional/local level investigated with respect to how concerns for genetic biodiversity are expressed. The color-coded DNA symbols indicate the average degree to which conservation genetic concerns are included in the documents—*green* good, *yellow* insufficient, *red* nothing/poor (cf. Tables 1, 2, 3, S1)

We first identified key international agreements and regulations that apply to the Baltic Sea including its biodiversity: two conventions and four EU directives (Fig. 1). The Convention on Biological Diversity (CBD; www.cbd.int) is global and overriding, whereas the Convention on the Protection of the Marine Environment of the Baltic Sea Area (the Helsinki Convention; www.helcom.fi) focus directly on the Baltic Sea. Similarly, the EU Habitats Directive (Directive 92/43/EEC), the EU Birds Directive (Directive 2009/147/EC), the EU Marine Strategy Framework Directive (MSFD; Directive 2008/56/EC), and the EU Water Framework Directive (WFD; Directive 2000/60/EC) have a regional focus and should, with respect to biodiversity, reflect implementation of the CBD within the EU.

We reviewed how concerns regarding gene level biodiversity are formulated in these six main documents, as well as in a total of 49 identified follow-up agreements and guidelines (Fig. 1; Table 1). The follow-up documents represent guidelines, strategies, recommendations, etc. that have been elaborated to guide national implementation of the main agreement at the international level. We chose to analyze only a subsample of all available such documents and selected a sample which appeared to be of relevance for biodiversity and investigated these documents with respect to genetic biodiversity.

Next, we addressed how these policies were implemented at the national level by reviewing national policy documents and the national reports to the institutions of the identified international agreements, including the secretariats of international conventions and to the Commission of the European Union (EU). We were able to focus on a subset of four countries and chose Sweden, Finland, Estonia, and Germany because (i) together they cover a large part of the Baltic Sea coastline (c. ¾), thus conservation practices within these countries have a large influence over the Baltic area, (ii) they represent regional variation by including northern, eastern, as well as central European countries, and (iii) they represent early, moderate, and late memberships of the EU (Germany 1957, Sweden and Finland 1995, Estonia 2004).

We chose to include six documents per country reflecting examples of national implementation of the international policies. We focused on a subset for which we were able to obtain comparable documents from all four countries. The selected documents include (i) national strategies for conservation of biodiversity that could be obtained from government official webpages or via email from government ministry officials, (ii) national biodiversity and action plans reflecting national implementation of the CBD and EU directives, (iii) the fifth national reports to the CBD (ii and iii obtained from www.cbd.int, October 2014), (iv) national implementation plans for the HELCOM Baltic Sea Action Plan (obtained from www.helcom.fi, October 2014), and (v) country specific technical assessment reports generated by the EU Commission to monitor MSFD and WFD implementation (http://ec.europa.eu/environment/water/water-framework/impl_reports.htm; http://ec.europa.eu/environment/marine/eu-coast-and-marine-policy/implementation/reports_en.htm, accessed October 2014).

**Fig. 2:**
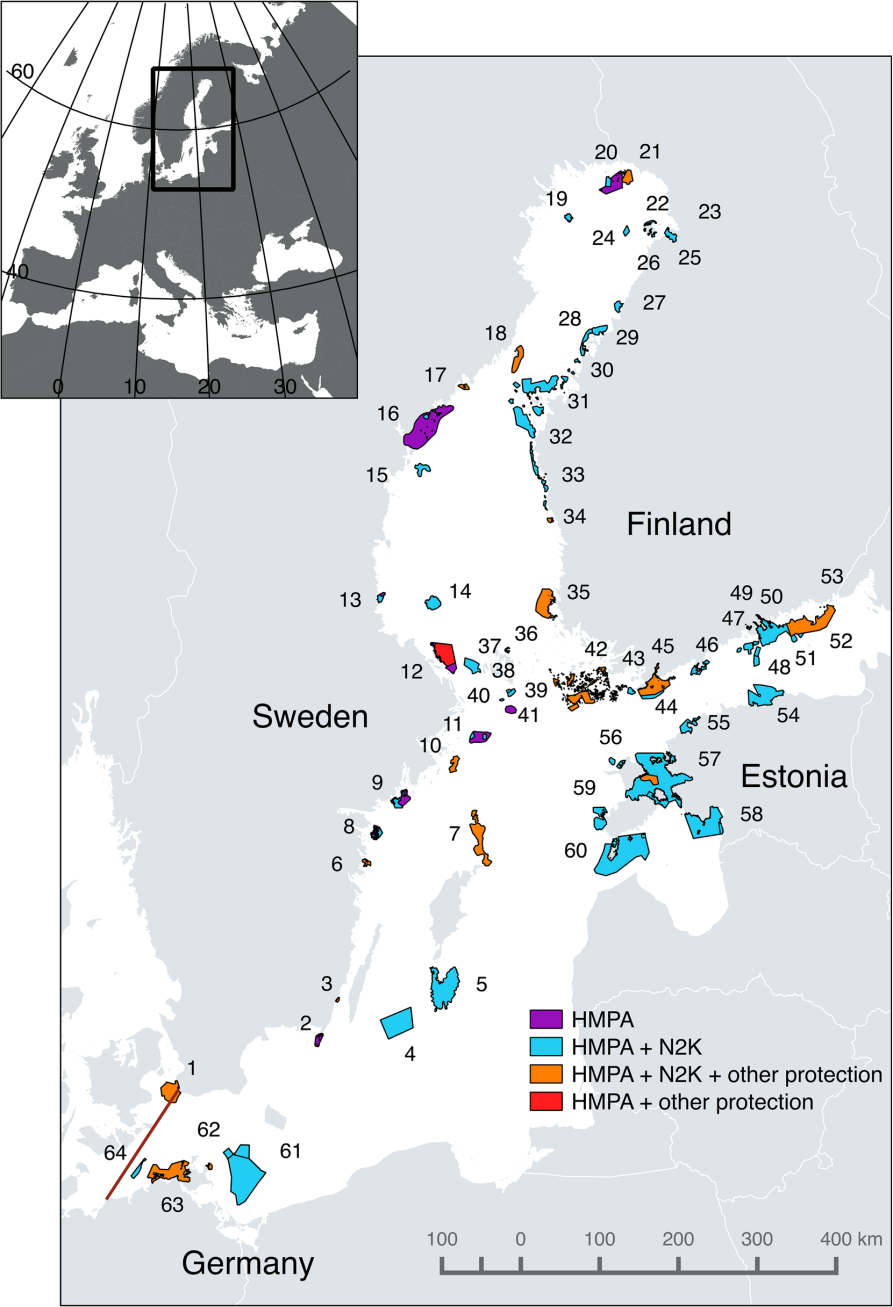
Map of Baltic Sea area showing the border we used to define the Baltic Sea for the purpose of this study (cf. Johannesson and André 2006). The 64 HELCOM MPAs of Sweden (20), Finland (33), Estonia (7), and Germany (4) are indicated as colored areas. Each HELCOM MPA has been numbered and further information on each area can be found in Tables 3 and S1. Different colors indicate the type of overlap (if any) with other types of protection for part or whole of the same area. *HMPA* HELCOM MPA, *N2K* Natura 2000. Other protection includes nature reserves and national parks

In the final step, we investigated how international and national policies concerning genetic biodiversity are implemented at the regional/local level focusing on Marine Protected Areas (MPAs) in the Baltic Sea in the four countries. There are several types of MPAs in the Baltic Sea, both international and national ones, and after becoming aware of considerable complexity with respect to the management structure (Appendix S1) we decided to focus on HELCOM MPAs that represent regional implementation of the Helsinki Convention (HELCOM Recommendations 15/5 and 35/1; Table 1). We used the Johannesson and André (2006) definition of the Baltic Sea entrance (Fig. 2) and collected all management plans that we were able to locate for HELCOM MPAs of the four countries in the defined area. Finding management plans was not straightforward in any of the countries and we had to use Internet searches, email correspondence, as well as many telephone contacts.

**Fig. 3:**
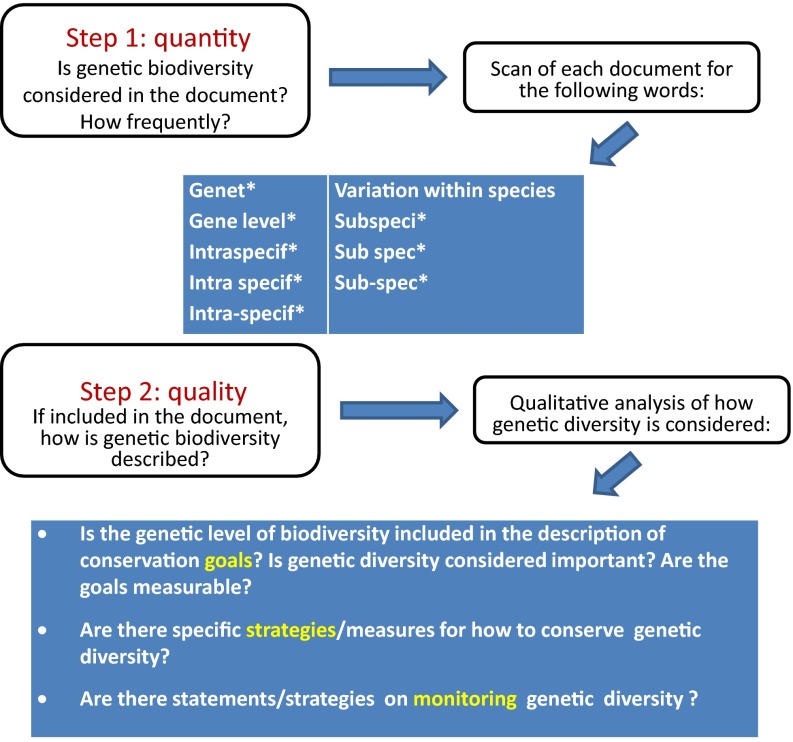
The textual analyses of the compiled documents followed the steps outlined here. In step 1, each document was scanned manually for the listed search terms and the number of times these words were found (number of hits) was used in quantitative analyses. In step 2, the text located by the hits was analyzed qualitatively using the listed guiding questions

In total, we analyzed 240 documents with 55 of them representing the international level, 24 the national level, and 161 the regional level of Baltic Sea MPAs (Fig. 1). We performed quantitative and qualitative textual analyses of these documents following the steps and using the search terms shown in Fig. 3. In cases where the documents were not available in English we used appropriate translations of keywords based on consultations with native speakers of each country.

The quantitative analyses included evaluating potential differences between countries and/or types of international agreements with respect to the number of times our search words (cf. Fig. 3) occurred in each document. To obtain a relative measure of occurrence, we related the number of hit words in a document to (i) the total number of words in the document and (ii) to the total number of pages in the document, thus obtaining two measures of frequency of search words per document. For the MPA management plans we quantified number of hits per plan. We then evaluated potential differences in the frequency at which the search terms occurred from the separate countries and between types of documents by means of analyses of variance tests (single- and two-factor ANOVAs) performed with MS Excel and exact Chi-square tests performed with StatXact v. 3.1. The statistical testing was performed when the documents analyzed could be regarded as a sample of a larger population of documents, as in the case of the follow-up documents, the documents at the national level, and the management plans. The six main international agreements, however, were not treated as a sample as we have included all international agreements that apply to Baltic Sea biological diversity (thus, these documents represent the true population).

The qualitative analysis included evaluating the text located by the search words to find out what was expressed concerning genetic variation. This included finding out if a separate document expressed conservation goals for genetic variation and whether these goals were measurable, encompassed strategies for how to conserve genetic variation, and whether means for monitoring and evaluating genetic variation was included.

## Results

### International level

The quantitative assessment shows that there is a clear difference among the six main international documents (two conventions and four EU directives; Fig. 1) with respect to the amount of times genetic biodiversity is mentioned. In the Convention on Biological Diversity (CBD), we find our search words 26 times, while they occur three times in the EU Habitats Directive, once in the EU Marine Strategy Framework Directive, and no times at all in the EU Water Framework Directive, the Helsinki Convention, and the EU Birds Directive (Table 1).

**Table 1:** International agreements and policy documents relating to biological diversity of the Baltic Sea analyzed in this study. No hits = number of times any of the search words referring to the genetic level of diversity were found in the document (cf. Fig. 3), words = number of words in document, pages = number of pages in document. A brief summary of the statements concerning genetic variation relating to the questions of Fig. 3: 1. Does the document include conservation goal(s) for genetic diversity? 2. Does the document include strategies for genetic conservation? 3. Does the document include statements/strategies for monitoring genetic diversity?

Agreement	No. hits, words, pages	Brief summary of statements concerning genetic biodiversity
International conventions		
United Nations Convention on Biological Diversity (CBD 1993)	26 9440 28	Conservation of biodiversity of ecosystems, species, and genes a fundamental goal. Genetic resources (= genetic material of actual or potential value) are highlighted as being of utmost importance. 1. Yes, high priority, not measurable. 2. Yes, but linked to biological diversity (e.g., legislation, area protection, research and transfer of technology). 3. Yes (genetics as part of biological diversity)
Convention on the Protection of the Marine Environment of the Baltic Sea Area (Helsinki Convention 1992, entered into force 2000)	0 13 033 26	Nothing relating to genetic diversity
EU directives		
The Habitats Directive (Council Directive 92/43/EEC)	3 14 454 44	The Natura 2000 network is of vital importance for “…the migration, dispersal and genetic exchange of wild species…” (Article 10). 1. Yes, high priority. 2. Yes, mainly area protection. 3. Not explicitly linked to the gene level
The Water Framework Directive (Directive 2000/60/EC)	0 33 714 82	Nothing relating to genetic diversity
The Birds Directive (Directive 2009/147/EC)	0 5737 25	Conservation of listed populations. The gene level is not explicitly referred to
The Marine Strategy Framework Directive (Directive 2008/56/EC)	1 12 598 22	1. Yes, the genetic level is one of several indicators to be used when determining environmental status of marine areas. 2. Calls for inventories of genetically distinct forms of native species. Protected areas need to meet the requirements in the CBD (the gene level is not explicitly mentioned). 3. See question 2
Follow-up documents to the conventions		
CBD (2 documents)		
Strategic Plan for Biodiversity 2011-2020 and the Aichi Biodiversity Targets (COP10 Decision X/2, 2010)	14 7210 13	Strategic goal C: “Improve the status of biodiversity by safeguarding ecosystems, species and genetic diversity.” Target 13: “By 2020, the genetic diversity of cultivated plants and farmed and domesticated animals and of wild relatives, including other socio-economically as well as culturally valuable species, is maintained, and strategies have been developed and implemented for minimizing genetic erosion and safeguarding their genetic diversity”. 1. Yes, high priority. 2. Yes, but unspecified (except for biodiversity—protected areas). 3. Not explicitly linked to the gene level
Consolidated update of the Global Strategy for Plant Conservation 2011-2020 (COP10 Decision X/17, 2010)	5 3046 7	Mentions endurance of plant genetic diversity. Target 5: “At least 75 per cent of the most important areas for plant diversity of each ecological region protected with effective management in place for conserving plants and their genetic diversity.” Target 9: “70 per cent of the genetic diversity of crops including their wild relatives and other socio-economically valuable plant species conserved…” 1. Yes, high priority (a sustainable future presupposes genetic diversity). Some goals are measurable (above). 2. Yes, protection of important areas and the genetic diversity of crops. 3. Yes, implicitly
HELCOM (16 documents)		
Recommendation 15/1, Protection of the coastal strip (1994)	0 939 2	Coastal areas important for biodiversity. CBD is mentioned but not genetic diversity
Recommendation 15/5, System of coastal and marine Baltic Sea Protected Areas (1994)	0 1357 5	Protection of representative ecosystems. Refers to CBD. Genetic diversity is not explicitly considered
Recommendation 16/3, Preservation of natural coastal dynamics (1995)	0 691 2	Preservation of biodiversity in coastal areas. The genetic level of biodiversity is not explicitly considered
Recommendation 17/2, Protection of harbour porpoise in the Baltic Sea area (adopted 1996, revised 2013)	0 414 1	Concern about the status of harbour porpoise in the Baltic Sea. The genetic level is not mentioned
Recommendation 18/4, Managing wetlands and freshwater ecosystems for retention of nutrients (1997)	0 470 2	Nothing relating to genetic diversity
Recommendation 19/2, Protection and improvement of the wild salmon (*Salmo salar L.*) populations in the Baltic Sea area (1998)	4 941 3	Genetic diversity is vital to the survival of wild salmon populations. Goal to attain “safe genetic limits,” calls for “immediate actions” to safeguard salmon survival and genetic diversity. 1. Yes, high priority. 2. Yes, immediate action is called for. 3. Yes. “The releases of reared salmon should be carefully monitored and their genetic or other impact on wild salmon evaluated by scientists”
Recommendation 19/3, Manual for the marine monitoring in the COMBINE programme of HELCOM (1998)	0 473 2	Nothing relating to genetic diversity
Recommendation 27-28/2, Conservation of seals in the Baltic Sea area (2006)	0 1399 3	Nothing relating to genetic diversity
Planning and Management of Baltic Sea Protected Areas: guidelines and tools (Baltic Sea Environment Proceedings, No 105; HELCOM 2006)	6 46 263 88	Genetic diversity important conservation and management goal. Can be attained through MPAs. 1. Yes. 2. Yes, “preserve genetic diversity” though area protection. 3. Not explicitly linked to the gene level
Baltic Sea Action Plan (2007)	4 36 949 101	Goal: favorable conservation status of Baltic Sea biodiversity in line with CBD. “Genetic variability” and “safe genetic limits” stressed as important goals for salmon, sea trout, and sturgeon. 1. Yes, genetic variability and safe genetic limits for salmon, sturgeon, trout. 2. Appropriate breeding and re-stocking practices in place by 2012. 3. No, but inventory and classification of Baltic salmon rivers
Toward an ecologically coherent network of well-managed Marine Protected Areas–Implementation report on the status and ecological coherence of the HELCOM BSPA network (HELCOM 2010, Baltic Sea Environment Proceedings, No 124B)	5 58 518 146	Conservation of genetic diversity an overarching objective. Criteria to be used when evaluating the BSPA network: “…connectivity among protected areas is of vital importance. It … allows for genetic interchange between populations.” 1. Yes, a general aim of MPAs is to protect genetic diversity. 2. Yes, via MPAs and connectivity among them. 3. Genetic diversity and the connectivity among protected areas are important to consider when evaluating the ecological status and coherence of the MPAs
Recommendation 32-33/1, Conservation of Baltic salmon (*Salmo salar*) and sea trout (*Salmo trutta*) populations by the restoration of their river habitats and management of river fisheries (2011)	1 2058 6	Genetic diversity addressed for stocking practices; “stocking for enhancement purposes is conducted on a temporary basis until natural reproduction reaches stable levels and are based on original strains or if not available on nearby populations with genetic proximity and similar ecological conditions.” 1. Yes, implicitly. 2. Yes, with regard to stocking practices. 3. Yes, implicitly
Recommendation 34E/1, Safeguarding important bird habitats and migration routes in the Baltic Sea from negative effects on wind and wave energy production at sea (2013)	0 1277 4	Nothing relating to genetic diversity
Taking Further Action to Implement The Baltic Sea Action Plan–Reaching Good Environmental Status for a healthy Baltic Sea (Ministerial Declaration 2013)	2 10 938 20	Genetics addressed for sustainable aquaculture, to prohibit risks of “ecological and genetic impacts on wild fish stocks from unintended releases of farmed species,” and concerning conservation of Baltic salmon and sea trout; genetic guidelines needed to improve stocking practices. 1. Yes, for sustainable aquaculture and conservation of salmon and sea trout. 2. Yes (genetic guidelines). 3. Yes, implicitly
Overview of implementation of the HELCOM Baltic Sea Action Plan (2013)	1 18 681 40	Subspecies are mentioned with reference the HELCOM Red List of Baltic Sea Species. 1. Yes, below species diversity in terms of subspecies. 2. Yes, protection of subspecies. 3. Yes, implicitly
Recommendation 35/1, System of coastal and marine Baltic Sea protected areas (2014)	1 2248 5	Genetic diversity is recognized as one of the Aichi targets that need to be reached. 1. Yes. 2. Yes, protected areas and “other effective area-based conservation measures.” 3. Not explicitly linked to the gene level of biodiversity
Follow-up documents to the EU directives		
The Habitats and Birds Directives (4 documents)		
Communication from the Commission to the Council and the European Parliament—Biodiversity Action Plan for Economic and Development co-operation (COM/2001/0162 final)	16 8969 11	Genetic resources important. Loss of genetic diversity in agriculture a large problem. Refers to CBD. 1. Yes, high priority. 2. Policies, investments, research, gene banks, protected areas. Protected areas in representative habitats and areas of high diversity maintain genetic resources. 3. Yes, “careful assessment of the most useful/important species/populations. Wild relatives of domestic stocks should be included in these assessments”
Guidelines for the establishment of the Natura 2000 network in the marine environment. Application of the Habitats and Birds Directives (2007)	8 46 622 112	Genetic diversity a rationale for site selection; isolated populations tend to contribute stronger to genetic diversity of species. Genetics mentioned for bird inventories and effects of aquaculture; escapes of individuals that are genetically different can affect local populations. 1. Yes, degree of isolation of population is “an approximate measure of the contribution of a given population to the genetic diversity of the species and of the fragility of the specific population at the site being considered.” 2. Yes, mainly area protection. 3. Yes, concerning isolated populations and inventories of rare bird subspecies
Our life insurance, our natural capital: An EU biodiversity strategy to 2020 (European Parliament resolution 2012)	8 8653 20	The Commission is called upon to develop a strategy for the conservation of genetic diversity. Genetics important in agriculture, for human, and animal sustenance. Mentions Aichi targets. 1. Yes, high priority. 2. More research on genetics. 3. Implied rather than explicitly stated with regard to the gene level of biodiversity
Assessment and reporting under Article 17 of the Habitats Directive. Explanatory Notes & Guidelines for the period 2007-2012 (2011)	18 44 923 123	Genetic structure of species should be considered when estimating its conservation status. Genetic variability is included when assessing the quality of a habitat. “Genetic pollution” is mentioned as a threat resulting from release of non-native conspecifics. 1. Yes. 2. Yes, include genetics when assessing species and habitat conservation status. 3. Implicitly as assessments should be carried out and reported continuously
The Marine Strategy Framework Directive (2)		
Commission Decision of September 01, 2010 on criteria and methodological standards on good environmental status of marine waters	5 7270 11	Genetic structure relevant to consider when estimating conservation status of species and habitats. 1. Yes. 2. Consider genetics when assessing species and environmental status. 3. Not explicitly
Action Plan for the European Union Strategy for the Baltic Sea Region (EU Commission 2013)	11 66 884 191	Genetic resources high priority but primarily for agriculture and forestry. 1. Yes, genetic variation is important for food, forestry, and agriculture. 2. Cooperation networks, information exchange/education, a European database on plant genetic resources. 3. See strategies
The Water Framework Directive (25)		
Common Implementation Strategy for the Water Framework Directive (2000/60/EC). Guidance Documents (GD) No 1-3, 5-9, 11-14, 17, 20-27, 30-33	7 995 541 2660*	GD No 3 (1 hit): refers to the threat imposed by genetic contamination of wild fish populations. GD No 12 (1): quotes the text of the Habitats Directive on genetic exchange of wild species. GD No 25 (2): concerns experimental methodology when monitoring chemicals in biota and the possibility to reduce unwanted effects of genetic differences among sampling organism. GD No 27 (3): concern that certain chemicals can cause genetic effects, and that genetic differences among model organisms for chemical testing can affect the results

* Total for all 25 documents

In the follow-up documents to the six main documents, we find increasing occurrence of genetic search words as compared to the main document (Table 1). For instance, guidelines and action plans following the Helsinki convention mention genetic biodiversity, whereas the main document does not.

When we grouped our sample of 49 follow-up documents after the six main conventions/directives, we found a statistically significant difference in the occurrence of genetic search words among the six groups of documents. This difference is observed regardless of whether we measure hits per words in document (measured as per mille hit words compared to total word count; single-factor ANOVA gives *F*_4,44_ = 4.68, *P* = 0.003) or as hits per page in document (*F*_4,44_ = 8.13, *P* ≪ 0.001). The difference disappears, however, when the follow-up documents of the Water Framework Directive (WFD) are removed from the analysis. This indicates that low occurrence of genetic search terms in WFD documents explains the difference among follow-up documents grouped after main international agreement.

When grouping and comparing follow-up documents to agreements that focus on the aquatic environment specifically (i.e., Helsinki Convention, Marine Strategy Framework Directive, and Water Framework Directive) versus those with a broader focus (CBD, Habitats Directive, and including the Birds Directive in this second group), we found a strongly significant statistically lower frequency of genetic search words in the group of aquatic documents for both types of measurements (*F*_1,47_ = 11.56, *P* = 0.001, and *F*_1,47_ = 24.83, *P* ≪ 0.001, for per mille hits per words and hits per page, respectively). This difference remains also when removing the WFD follow-up documents when measuring hits per page (*F*_1,22_ = 7.89, *P* = 0.010) but not when measuring per mille hits per word (*F*_1,22_ = 3.07, *P* = 0.093). Thus, the low occurrence of genetic diversity in documents focusing on the aquatic environment is not explained fully by low occurrence in WFD documents. Rather, other aquatic follow-up documents (MSFD and Helsinki Convention) appear to have low mentioning of genetics in comparison to the broader focused ones (follow-up to CBD, Habitats and Birds Directives; cf. Table 1).

#### Conservation goals

The qualitative textual analysis shows that the genetic level of biological diversity is recognized as a conservation goal in three of the six main documents at international level. The CBD states that genetic diversity is a key component of biodiversity and the Habitats Directive clearly stipulates the importance of intraspecific variation in conservation. The Marine Strategy Framework Directive (MSFD) mentions genetic diversity as one of several indicators to be used in determination of environmental status. The Helsinki Convention, the WFD, and the Birds Directive do not mention genetic variation as a conservation goal.

In 20 of the 49 follow-up documents, concerns regarding genetic variation are mentioned, and in 15 of these documents, conservation goals for genetic diversity are expressed (Table 1). Such goals are strongly stated in the EU Biodiversity Strategy to 2020 and the EU Guidelines for establishing Natura 2000 network in the marine environment, which concerns EU implementation of, e.g., the Habitats and Birds Directives, as well as in the CBD Strategic Plan 2011-2020 including Aichi Target 13 directly focusing on genetic biodiversity (COP10 Decision X/2, 2010; www.cbd.int/sp/targets/; Table 1).

Genetic variation is mentioned as a conservation goal in follow-up documents also to those main documents that do not mention genetic diversity. The only exception is the Water Framework Directive—we could find a few references to the intraspecific level of biodiversity in four of the 25 documents analyzed but no clear goals were expressed.

The Helsinki Convention does not mention genetic biodiversity in the main document, but a recommendation from 1998 stresses that genetic diversity is crucial to the survival of Baltic salmon (HELCOM Recommendation 19/2). In later HELCOM documents, genetic diversity is mentioned as an important conservation goal and MPAs are described as important means for reaching this goal (Table 1).

#### Strategies, measurable goals, and monitoring

International goals on genetic diversity are typically not expressed in measurable terms. An exception is the Global Strategy for Plant Conservation (CBD COP10 Decision X/17) where the Target 9 goal for 2020 says: “70 % of the genetic diversity of crops including their wild relatives and other socio-economically valuable plant species conserved.”

Area protection is the most common strategy to conserve genetic diversity; “[e]stablish a system of protected areas or areas where special measures need to be taken to conserve biological diversity [genetic resources included]…” (CBD 1993, Article 8). The Natura 2000 network and “their linear and continuous structures…” are of vital importance for “…the migration, dispersal and genetic exchange of wild species…” (The Habitats Directive, 1992, Article 10). Other strategies include legislation, policies, research, inventories, databases, gene banks, breeding and re-stocking, habitat restoration, technology and information exchange (Table 1).

Monitoring is seldom explicitly linked to the gene level, but the CBD (1993, Article 7) states that contracting parties “shall, as far as possible and as appropriate…” “[i]dentify components of biological diversity important for its conservation and sustainable use…” and “[m]onitor, through sampling and other techniques, the components of biological diversity…” identifying biological diversity as variation of ecosystems, species, and genes. An explicit call for genetic monitoring in the Baltic Sea concerns protection of wild salmon (*Salmo salar*): “releases of reared salmon should be carefully monitored and their genetic or other impact on wild salmon evaluated by scientists” (HELCOM Recommendation 19/2; Table 1).

### National level

The quantitative analysis indicates a trend of difference among the four countries with respect to the occurrence of search terms in national implementation documents (Table 2) which is statistically significant when measuring number of hits per page (*F*_3,16_ = 3.66, *P* = 0.035), but not fully so when measuring hits per word in documents (per mille hit words per word in document; *F*_3,16_ = 2.61, *P* = 0.087). The highest frequency occurs in Finnish documents and the lowest in Estonian ones (average number of hits per page over the six documents is 0.69 for Finland vs. 0.16 for Estonia). Also, we observe a difference between types of documents when we compare reporting documents relating to the CBD and the EU Habitats and Birds Directives versus those with a marine/aquatic focus (Helsinki Convention, Marine Strategy Framework Directive, and Water Framework Directive) that occur both when measuring per mille hits per words in documents (*F*_1,16_ = 34.42, *P* < 0.001) and hits per page in documents (*F*_1,16_ = 41.23, *P* ≪ 0.001). Here, average number of hits per word over documents and countries are 1.83 for CBD-, Habitats-, and Birds-Directive-related ones versus 0.12 for the marine policy-related documents, whereas average number of hits per page gives 0.82 versus 0.06 for the same comparison (CBD- vs. marine-related policy documents; cf. Table 2).

**Table 2:** National policy documents exemplifying implementation of international agreements applying to conservation of gene level biodiversity of Baltic Sea species (cf. Table 1). Brief summaries of how genetic variation is addressed in these documents are given (cf. Fig. 3 for analysis procedure). CBD = Convention on Biological Diversity, MSFD = Marine Strategy Framework Directive (EU Directive), WFD = Water Framework Directive (EU Directive), No hits = number of times search words for genetic diversity (cf. Fig. 3) occur in document

International agreement	National document	Country			
		Sweden	Finland	Estonia	Germany
CBD, EU Habitats, and Birds Directives	Document	A Swedish Strategy for Biological Diversity and Ecosystem Services. Government Bill 2013/14:141 (Swedish Government 2013; In Swedish)	Government Resolution on the Strategy for the Conservation and Sustainable Use of Biodiversity in Finland for the years 2012–2020, ‘Saving Nature for People’ (Finnish Government 2012)	Estonian Nature Conservation in 2011 (Estonian Environment Information Centre 2012)	National Strategy on Biological Diversity (Federal Ministry for the Environment, Nature Conservation, Building and Nuclear Safety 2007)
	No hits	88 (72870 words, 192 pages) 1.2 ‰ hits/word, 0.46 hits/page	51 (11633 words, 26 pages) 4.4 ‰/word, 1.96 hits/page	13 (44136 words, 126 pages) 0.3 ‰/word, 0.10 hits/page	247 (100312 words, 180 pages) 2.5 ‰/word, 1.37 hits/page
	Summary statements on genetic diversity	Genetic diversity is important for maintaining viable populations of species and to ensure the resilience of ecosystems. **Strategies:** Mapping and **monitoring** genetic variation in wild and domesticated plants and animals needed and started by 2015. **Goal:** Aichi targets recognized	**Goal:** Commitment to CBD objectives, including conservation and sustainable use of biodiversity. **Strategies**: ex situ conservation projects to support in situ conservation. **Monitoring** trends in genetic resources for agriculture and forestry	Genetic diversity a vital part of biodiversity. **Goal:** Genetic erosion in cultivated plants, forestry, agriculture, farmed/domesticated animals and wild relatives needs preventing. **Strategies** are called for but not specified, except coastal/marine area protection	**Goal**: maintain genetic diversity and natural distribution of species in Germany. Genetically distinct populations conserved. Loss of genetic diversity halted by 2010. Area protection main **strategy** to protect genetic diversity in nature
CBD	Document	Information on the Swedish national biodiversity strategies and action plans (Ministry of Sustainable Development 2006)	Saving Nature for People. National action plan for the conservation and sustainable use of biodiversity in Finland 2013-2020 (Finnish Government 2012)	Nature Conservation Development Plan until 2020 (Ministry of the Environment 2012)	National Strategy on Biological Diversity (the German Cabinet 2007)
	No hits	126 (95839 words, 236 pages) 1.3 ‰/word, 0.53 hits/page	152 (66273 words, 107 pages) 2.3 ‰/word, 1.42 hits/page	27 (24475 words, 54 pages) 1.1 ‰/word, 0.50 hits/page	247 (65644 words, 242 pages) 3.8 ‰/word, 1.02 hits/page
	Summary statements on genetic diversity	**Goal** to conserve genetic diversity explicit. **Strategies**: conservation of ecosystems and viable populations. Points to difficulties to measure genetic variation. Calls for research and synthesis on genetic variation and marine biodiversity. Present knowledge-gaps make defining objectives and actions for to genetic diversity difficult	Genetic diversity incl. in **goal**. Identifies: genetics insufficiently included in environmental impact assessments. **Strategies:** protect species and habitats, measures against alien species, live gene bank for fishes, agriculture, and forestry. **Monitoring** of genetic diversity of fish stocks shall increase	**Goal**: highest possible level of genetic diversity maintained Genetic profiles of subspecies/populations to be conserved. Genetic diversity of salmon, brown trout, asp, Atlantic sturgeon, European cat/crayfish threatened. **Strategies**: gene banks, avoiding spread of alien species and GMOs	**Goal**: loss of genetic diversity halted by 2010. Vision: conserve genetic variation of wild animals and plants in Germany including area-typical populations for ability to adapt to changing environments. **Strategies**: area protection and use of the precautionary principle
CBD	Document	Fifth National Report to the CBD—Sweden (Swedish Government 2014)	Fifth National Report to the CBD—Finland (Ministry of the Environment 2014)	V National Report to the CBD (Estonian Ministry of the Environment 2014)	Fifth National Report, CBD (Federal Ministry for the Environment, Nature Conservation, Building and Nuclear Safety 2014)
	No hits	60 (35824 words, 77 pages) 1.7 ‰/word, 0.78 hits/page	97 (70663 words, 141 pages) 1.4 ‰/word, 0.69 hits/page	19 (41346 words, 86 pages) 0.5 ‰/word, 0.22 hits/page	99 (59071 words, 131 pages) 1.7 ‰/word, 0.76 hits/page
	Summary statements on genetic diversity	Genetic diversity objectives related to CBD targets. Milestone target: mapping and **monitoring** of genetic diversity are initiated by 2015. Notes: Swedish 16th environmental quality objective *A Rich Diversity of Plant and Animal Life* lack indicators that measure genetic variation. A draft action plan for protection of genetic variation in wild species exists, but not yet implemented	Relate to Target 13; genetic biodiversity of cultivated plants and wild relatives, forest trees, fish stocks, farmed/domesticated animals safeguarded by 2020 “Enhance the **monitoring** of genetic diversity of fish stocks and their sub-stocks.” Calls for research and a national program for knowledge and awareness on the importance of plant genetic resources called for	Preservation of genetic diversity important for biodiversity. Focus is on agriculture. A plan for the collection of plant genetic resources is called for	Stresses importance of genetic variation for species survival and adaptation. Protect genetic wild population genetic diversity from harmful effects of alien species and “breeding varieties.” Genetic exchange among marine populations necessary with inter-linked marine biotopes. **Strategies**: research, genetic studies of endangered species, information, conservation networks
Helsinki Convention	Document	National Implementation Plan for the Baltic Sea Action Plan (Government Offices of Sweden 2010)	Implementation of HELCOM’s Baltic Sea Action Plan (BSAP) in Finland. Status Report 17 May 2010 (The Ministry of the Environment, Finland)	Baltic Sea Action Plan Implementation Programme 2008–2011 (Estonian Government 2008)	Implementation of the HELCOM Baltic Sea Action Plan (BSAP) in Germany (German Government 2011)
	No hits	1 (15603 words, 28 pages) 0.06 ‰/word, 0.04 hits/page	0 (10576 words, 16 pages) 0 ‰/word, 0 hits/page	3 (11362 words, 27 pages) 0.26 ‰/word, 0.11 hits/page	0 (29640 words, 87 pages) 0 ‰/word, 0 hits/page
	Summary statements on genetic diversity	Gene level mentioned with regard to a “small, genetically isolated population of around 200 harbour porpoise”	The genetic level is not explicitly considered	The gene level is addressed for preservation of salmonid populations. Calls for maintaining genetic diversity in artificial breeding and release of sea trout. Genetic mixing of geographically separate populations should be avoided	The genetic level is not explicitly considered
MSFD	Document	Article 12 Technical Assessment of the MSFD 2012 obligations Sweden (2014)	Article 12 Technical Assessments of the MSFD 2012 obligations Finland (2014)	Article 12 Technical Assessments of the MSFD 2012 obligations Estonia (2014)	Article 12 Technical Assessments of the MSFD 2012 obligations Germany (2014)
	No hits	27 (26727 words, 51 pages) 1.0 ‰/word, 0.53 hits/page	3 (21019 words, 45 pages) 0.1 ‰/word, 0.07 hits/page	0 (15561 words, 38 pages) 0 hits/word, 0 hits/page	0 (28918 words, 53 pages) 0 hits/word, 0 hits/page
	Summary statements on genetic diversity	The reporting sheets list nine genetically distinct forms of native species for the Baltic region considered to be under pressure	“Good Environmental Status” includes sufficiently complex population genetic structure to allow adaptation to environmental change. **Goal**: preserve genetic diversity of sea trout. **Strategies**: restore streams to allow large spawning populations minimizing genetic change	The genetic level is not explicitly considered	The genetic level is not explicitly considered
WFD	Document	Member State: Sweden on the Implementation of the Water Framework Directive (2000/60/EC). River Basin Management Plans (European Commission 2012)	Member State: Finland on the Implementation of the Water Framework Directive (2000/60/EC). River Basin Management Plans (European Commission 2012)	Member State: Estonia on the Implementation of the Water Framework Directive (2000/60/EC). River Basin Management Plans (European Commission 2012)	Member State: Germany on the Implementation of the Water Framework Directive (2000/60/EC). River Basin Management Plans (European Commission 2012)
	No hits	0 (19370 words, 63 pages) 0 hits/word, 0 hits/page	0 (22106 words, 64 pages) 0 hits/word, 0 hits/page	0 (16124 words, 50 pages) 0 hits/word, 0 hits/page	0 (31078 words, 87 pages) 0 hits/word, 0 hits/page
	Summary statements on genetic diversity	The genetic level is not explicitly considered	The genetic level is not explicitly considered	The genetic level is not explicitly considered	The genetic level is not explicitly considered

Further, there is a significant interaction between country and type of document (measuring per mille hits/word gives *F*_3,16_ = 4.89, *P* = 0.013, and per page: *F*_3,16_ = 3.48, *P* = 0.041) suggesting that there is a difference with respect to how often the countries include genetic terms in CBD/EU Habitats and Birds Directives versus Helsinki Convention/EU Marine Strategy Framework Directive, and Water Framework Directive reporting documents; the highest frequency of genetics in marine/aquatic documents occurs in Sweden (28 hits in the three documents), and the lowest in Germany (0 hits in all three documents).

#### Conservation goals

The qualitative textual analysis shows that all countries recognize genetic diversity as an important component of biological diversity that is of conservation value in their national biodiversity strategies and action plans as well as in their fifth national reports to the CBD (Table 2). It is generally understood that genetic diversity is necessary for evolutionary adaptation to environmental changes and goals of conserving genetic biodiversity are expressed by all four countries. Sweden and Germany use stronger and clearer wordings with respect to the importance of genetic variation of wild animals and plants, than Finland and Estonia.

Text concerning genetic diversity is rare and weak in the follow-up documents relating directly to the aquatic environment, i.e., to the Helsinki Convention, the Marine Strategy Framework Directive (MSFD), and the Water Framework Directive (WFD; Table 2). Importance of genetic variation for a few species is mentioned by Sweden (harbor porpoise) and Estonia (salmonids) in their National Implementation Plans for the Baltic Sea Action Plan (Table 2). In the MSFD assessments, only Sweden and Finland mention genetic diversity. Finland states that genetic diversity is crucial to the definition of Good Environmental Status in the marine environment. Similarly, none of the countries refer to genetic biodiversity in the implementation report under the WFD that we reviewed.

#### Strategies, measurable goals, and monitoring

National goals for genetic diversity are expressed as “loss of genetic diversity has been halted by 2010” (Germany; Table 2) and “genetic biodiversity of Finland’s cultivated plants and their wild relatives, forest trees, fish stocks, and farmed and domesticated animals has been preserved and safeguarded” by 2020. The Estonian government has a goal stating that “[m]echanisms to ensure the genetic diversity of species have been developed and applied” by 2020 (Table 2). The Swedish Government has defined a milestone target that national mapping and monitoring of genetic diversity should be initiated by 2015, and also specifically stresses the need for better understanding of marine biodiversity including genetic diversity. Thus, similar to the international goals, national goals on genetic diversity are typically not expressed in measurable terms.

Strategies for maintaining genetic diversity include upholding healthy ecosystems and viable populations (Sweden, Finland) for instance through area protection (Germany), more research and compiling existing information (Sweden), by avoiding spread of alien species and GMOs (Estonia, Germany), and by creating and maintaining gene banks and other ex situ programs (Finland, Estonia).

### Regional level

There are a total of 64 HELCOM MPAs in the Baltic Sea countries we investigated; 20 Swedish ones, 33 Finnish, 7 Estonian, and 4 German (Fig. 2; Tables 3, S1). In all four countries, the 64 HELCOM MPAs overlap with other types of protection including Natura 2000, and national protection measures such as national parks or nature reserves. Overall, the 64 HELCOM MPAs include other types of protected areas with 1–35 such areas (average = 3) per HELCOM MPA. Management responsibility varies among countries and rests with regional, County Administrative Boards (Sweden), regional authorities (Finland), federal states (Germany), and the National Environmental Board (Estonia).

**Table 3:** Baltic Sea HELCOM MPAs in Sweden (20), Finland (33), Estonia (7), and Germany (4) included in this study (cf. Fig. 3). HMPA = HELCOM MPA, No. N2 K = Number of Natura 2000 areas included in the HMPA. No. add. MPA = number of additional protected areas in the HMPA. Cover HMPA=”Yes”/”No” implies whether available management plans cover the whole HMPA area or not. Hits = number of times search terms (cf. Fig. 2) occur in management plan(s), in parenthesis number of plans with hits. Last column summarize content on genetic diversity. *The same one management plan refers to these three HMPAs. **Within HPMA Eastern Gulf of Finland Archipelago and waters another HMPA (Pernajabay and Pernaja) is also included. ***Included in the drafted management plan for the Bothnian Sea National Park to which this HMPA is a part. ****Included in a management plan for a larger protected area called Kalajoki Coast (not a HMPA). See Table S1 for more details

HELCOM MPA	Figure 3 ID	No. N2 K	No. add. MPAs	No. management plans	Cover HMPA	Hits (plans)	Content on genetic diversity
Finland—33 HELCOM MPAs 14 management plans							
Hailuoto northshore	22	1	–	1*	Yes	0	–*
Isomatala–Maasyvänlahti	25	1	–	1*	Yes	*	See Hailuoto
Eastern Gulf of Finland Archipelago and waters	53	2	1**	1	Yes	0	–
Kirkkonummi Archipelago	46	1	–	0	–	–	–
Kirkkosalmi	26	1	–	1*	Yes	*	See Hailuoto
Kokkola Archipelago	29	1	–	1	Yes	0	–
Kristiinankaupunki Archipelago	33	1	–	0	–	–	–
Liminka Bay	23	1	–	0	–	–	–
Luoto Archipelago	28	1	–	1	Yes	0	–
Outer Bothnian Threshold Archipelago—The Quark	31	1	–	1 (draft)	Yes	0	–
Närpiö Archipelago	32	1	–	0	–	–	–
Oura Archipelago	34	1	1***	1 (draft)***	Yes	1 (1)	Concerns the introduced, non-native mouflon sheep on an island in the MPA: their population size should be large enough to maintain genetic diversity
Pernajabay and Pernaja Archipelago MPAs	50	1	**	1	Yes	0	–
Bothnian Bay National Park	21	1	1	1 (draft)	Yes	0	–
Porvoonjoki Estuary-Stensböle	49	1	–	1	Yes	0	–
Rahja Archipelago	27	1	–	1****	Yes	0	–
Saaristomeri—Archipelago Sea	42	1	1	1	Yes	3 (1)	Regarding hunting important that genetic diversity is maintained. Gene banks potential aid for rare plants and animals
Söderskär and Långören Archipelago	47	1	–	1 (draft)	Yes	0	–
Tammisaari and Hanko Archipelago and Pojo Bay MPA	45	1	1	1	Yes	0	–
Tulliniemi bird protection area	43	1	–	1 (draft)	Yes	0	
Uusikaarlepyy Archipelago	30	1	–		–	–	–
Uusikaupunki Archipelago	35	1	***	1 (draft)***	–	***	See Oura Archipelago
Länsiletto	52	1	–	0	–	–	–
Luodematalat	51	1	–	0	–	–	–
Merikalla	24	1	–	0	–	–	–
Björkör Islands	39	1	–	0	–	–	–
Boxö Islands	36	1	–	0	–	–	–
Långör-Östra Sundskär Islands	38	1	–	0	–	–	–
Signilskär-Märket Islands	37	1	–	0	–	–	–
Sea area south from Sandkallan	48	1	–	0	–	–	–
Open sea area southeast from Hanko	44	1	–	0	–	–	–
Lågskär Islands	40	1	–	0	–	–	–
Bogskär Islands	41	–	–	0	–	–	–
Germany—4 HELCOM MPAs, 2 management plans							
Kadetrinne	64	1	–	0	–	–	–
Pommersche Bucht-Rönnebank	61	4	–	0	–	–	–
Jasmund National Park	62	1	1	1	Yes	2 (1)	Area aim includes preserving genetic resources of species
Vorpommersche Boddenlandschaft National Park	63	5	1	1	Yes	3 (1)	Aim of area includes preserving genetic resources of species. Baltic marine fish differ from North Sea populations. Two types of herring within the Baltic—spring and autumn spawning
Estonia—7 HELCOM MPAs 13 management plans							
Hiiu Madala	56	3	–	1	No	0	–
Kura Kurk	60	6	1	4	No	0	–
Lahemaa	54	1	1	1	Yes	0	–
Pakri	55	1		1	Yes	0	–
Pärnu lahe	58	4		1	No	0	–
Väinameri	57	2	2	3	No	1 (1)	Assisted gene flow by pasturing cattle needed for disconnected marsh angelica (*Angelica palustris*) populations along seashores to avoid genetic impoverishment of the plant
Vilsandi	59	2	–	2	Yes	1 (1)	Inbreeding a factor potentially affecting natterjack toad (*Epidalea calamita*) viability
Sweden—20 HELCOM MPAs 132 management plans							
Hoburgs bank	5	1	–	1	Yes	0	–
Kopparstenarna/Gotska Sandön/Salvorev Area	7	1	1	2	Yes	1 (1)	Areas with no fishing allowed protect genetically valuable individuals of fish
Axmar	13	1	1	2	No	0	–
Finngrundet-östra banken	14	1	–	1	Yes	0	–
Northern Midsjöbanken	4	1	–	0		–	
Värnanäs Archipelago	3	1	1	2	Yes	1 (1)	Baltic harbor seal (*Phoca vitulina*) population genetically vulnerable to small population size. Genetically separate from Swedish west coast populations
Haparanda Archipelago	20	13	2	15	No	14 (13)	Risk of negative genetic effects due to isolated populations of Siberian primerose (*Primula nutans*; 13 plans) and bluntleaf sandwort (*Moehringia lateriflora;* 1 plan). The exact same text is repeated in 13 plans
Marakallen	19	1	–	1	Yes	0	–
Falsterbo Peninsula with Måkläppen	1	5	7	12	No	4 (1)	Risk of negative genetic effects due to small population size for harbor seal *Phoca vitulina*, and due to small and isolated populations of little grapefirn (*Botrychium simplex*)
Torhamns Archipelago (Blekinge arkipelag)	2	5	4	9	Yes	0	–
Bullerö-Bytta	10	1	4	5	Yes	0	–
Stora Nassa–Svenska Högarna	11	2	2	5	Yes	8 (3)	Genetic exchange among populations needed for long-term favourable status. Unclear if this goal is reached. Risk of negative genetic effects due to small, isolated/poorly connected subpopulations of little grapefern (*Botrychium simplex*) and northern crested newt (*Triturus cristatus*). Restoring pike (*Esox lucius*) populations through releases should be documented and monitored to avoid negative genetic effects and inbreeding
Fifång (Askö-Hartsö)	9	11	12	23	Yes	3 (3)	Fragmentation of habitats results in a general threat due to lack of gene flow between populations. An endemic subspecies of *Cakile maritima ssp. baltica* is protected in two areas
Gräsö–Singö Archipelago	12	5	2	7	No	10 (3)	Pool frog (*Rana lessonae*) representing the genetically distinct Swedish population important to conserve. Genetically distinct populations often occur at distribution edges. A special section on genetic diversity and resilience with several examples in one plan
Kronören	17	1	1	2	Yes	1 (1)	Risk of negative genetic effects due to isolation in the endangered four leaf mare´s tail (*Hippuris tetraphylla*)
The Holmö Islands	18	1	1	2	Yes	0	
High Coast	16	13	22	35	Yes	14 (2)	Section of text on threats posed by release of alien species, populations, and genes including risks for genetic changes including loss of genetic variation. Subspecies of conservation concern listed
Vänta litets grund	15	1		1	Yes	0	
Kvädöfjärden med Torrö	6	1	2	3	Yes	1 (1)	Protected areas important as gene banks to protect genetically distinct fish populations. Protected areas conserve fishes that carry genes for rapid growth
S:t Anna–Missjö Archipelago	8	2	2	4	Yes	4 (2)	Fragmented habitats result in a general threat due to lack of gene flow between populations. Ecosystem approach to be applied aimed at securing all components of ecosystems including genetic variation. Probably genetic adaptation developed by Baltic Sea species. Population concept defined as a genetically separate group of individuals

We were able to locate a total of 161 management plans that apply to 45 of the 64 HELCOM MPAs; 19 HELCOM MPAs lack management plans (1 in Sweden, 2 in Germany, and 16 in Finland, 11 out of which were established during 2014–2015). The management plans have typically not been developed for the HELCOM MPA but for other types of protection that apply to the whole or parts of the same area (Natura 2000, national parks, or nature reserves).

For 8 of the 45 HELCOM MPAs that have management plans the plans only cover part of the HELCOM MPA area (4 Estonian, 4 Swedish; Tables 3, S1). In all four countries, other Natura 2000 and other types of protected areas exist in the Baltic Sea in addition to those included in the 64 HELCOM MPAs. Thus, we regard the analyzed management plans as a sample from a pool of all plans for protected areas in the Baltic Sea for our quantitative statistical assessments.

#### Genetic concerns in MPA management plans

Genetic concerns are rarely expressed in the management plans. In total we find 72 hits referring to genetic variation occurring in 37 out of 161 management plans (31 Swedish plans, 2 Estonian, 2 Finnish, and 2 German) representing 17 of the 64 HELCOM MPA areas. The frequency of plans that include genetics is thus 0.23, 0.14, 0.15, and 1.0 for Sweden, Finland, Estonia, and Germany, respectively, and close to a statistically significant difference among countries (Pearson’s *χ*^2^ = 7.74, *P* = 0.060).

The frequency of HELCOM MPAs with hits is 0.55, 0.06, 0.28, and 0.50 for Sweden, Finland, Estonia, and Germany, respectively, and is statistically different among countries (Pearson’s *χ*^2^ = 16.54, *P* < 0.001). The difference still holds when ignoring the 11 newly established Finnish HELCOM MPAs that all lack plans (Pearson’s *χ*^2^ = 10.79, *P* = 0.011), but not when removing Finland (which has considerably less hits than the other countries) altogether from the analysis. Thus, genetic concerns are significantly much less frequent for the Finnish HELCOM MPAs than for those of the other countries.

The genetic hits in management plans typically refer to concern for small population size and/or lack of gene flow in particular species; 33 of the 72 hits refer to such cases (Tables 3, S1). A total of 13 species are mentioned as having such genetic concerns, and of these 9 species are typically land or freshwater living (27 hits referring to fourleaf mare’s tail, pool frog, mouflon sheep (an introduced species), marsh angelica, natterjack toad, little grapefern, northern crested newt, bluntleaf sandwort, or Siberian primrose; Tables 3, S1) whereas only four are species whose primary habitat is the marine Baltic (6 hits referring to harbor seal, herring, or northern pike). Almost half of the Swedish hits (14 out of 31) refer to concern for genetic isolation of the Siberian primrose in Haparanda Archipelago.

General conservation goals for genetic biodiversity are expressed for very few HELCOM MPA areas (Table 3); Jasmund National Park, Vorpommersche Boddenlandschaft National Park (both in Germany), Stora Nassa–Svenska Högarna, and S:t Anna–Missjö Archipelago (in Sweden). Strategies for genetic conservation, either broadly for all species or for separate cases (cf. Table 3), include keeping the protected area as such (e.g., Kvädöfjärden med Torrö, Gräsö–Singö Archipelago, Sweden), maintaining gene flow to avoid isolated populations, including human-mediated gene flow (e.g., Väinameri, Estonia, Fifång, and Stora Nassa–Svenska Högarna, Sweden), avoiding fishing/hunting (Saaristomeri-Archipelago Sea, Finland, Kopparstenarna/Gotska Sandön/Salvorev Area and Kvädöfjärden med Torrö, Sweden), avoiding release of alien species, populations, or genes (High Coast, Sweden), and applying the ecosystem approach (S:t Anna–Missjö Archipelago, Sweden). Genetic monitoring is mentioned only in one case—with respect to restoration of pike populations through releases. Such releases should be monitored to avoid negative genetic effects (Stora Nassa–Svenska Högarna, Sweden).

#### A subsample comparison to species diversity

Genetic diversity is the focus of the present paper but for the purpose of discussion on how this level of biodiversity compares to the extent to which the species level of biodiversity is considered in the Baltic Sea MPA management plans we analyzed a subset of plans for species diversity. We searched all the 14 Finnish MPAs plans and 20 out of 132 Swedish plans for words relating to species and species diversity. These 34 plans represented the 17 Finnish and 19 Swedish HELCOM MPAs for which management plans are available. We found a total of 2714 hits reflecting species diversity in these 34 plans as compared to 30 hits for genetic search terms in the same plans. All of the 34 plans included species diversity hits (15–501 hits per plan) as compared to 10 of them containing hits for genetic search words (0–13 hits per plan); the difference is highly significant (paired *t* test gives *P* ≪ 0.001). Thus, genetic diversity appears to be considered much less than species diversity in Baltic Sea MPA management.

## Discussion

We conducted quantitative and qualitative textual analyses of 240 documents to investigate if and how concerns regarding genetic biodiversity expressed in international policies governing biological diversity in the Baltic Sea is transferred into national policy in Sweden, Finland, Estonia, and Germany. We then analyzed the extent to which expressed concerns, goals, and targets are further implemented in management plans of Baltic Sea MPAs in these four countries. Key findings are as follows:

1. International and national policy on genetic biodiversity are not reflected in management plans for marine protected areas of the Baltic Sea. Management plans in all four countries are largely void of goals, concerns, strategies, or other mentioning of genetic biodiversity.
2. Goals for genetic biodiversity are much less frequent in international and national policies directed exclusively toward aquatic environments (the Helsinki Convention and the EU Marine Strategy and Water Framework Directives) as compared to documents with a broader focus (CBD and the EU Habitats Directive).

Other results include the following:

3. International policy clearly express that genetic biodiversity should be conserved, strategies for such conservation should be formulated, and monitoring programs should be developed.
4. National policies in all four countries are in line with international intentions. Quantitatively, Finnish documents have the highest occurrence of our genetic search words, whereas qualitatively Swedish documents are strongest including most far-reaching intentions for monitoring genetic biodiversity of wild animals and plants.
5. Area protection is expressed as a frequent, explicit measure to conserve genetic biodiversity both at the international and the national level.
6. Genetic diversity is mentioned much less than species diversity in Baltic Sea MPA management plans.

The fact that the international policy that focus on aquatic environments in the Baltic Sea region is weak with respect to genetic diversity, and do not incorporate CBD conservation goals in the main documents, can to some extent explain the lack of conservation genetic concerns in MPA management plans. Such lag of conservation genetics in aquatic environments as compared to terrestrial ones was highlighted twenty years ago (Ryman et al. 1995) and obviously remains today. Several follow-up documents to the Helsinki Convention include CBD-related goals for genetic diversity and highlight the importance of marine protected areas for reaching such goals (Table 1). The documents at the national level that relate to this convention do not reflect this more recent inclusion of genetics, however (Table 2). Similarly, the national MSFD assessment documents do not include any genetic considerations in Estonia and Germany in spite of the MSFD main document listing genetic diversity as one indicator that should be considered for evaluating environmental status of marine areas (also underlined in follow-up documents to this directive; Table 1). Thus, the lag of including conservation goals and strategies for genetic biodiversity in documents at the international level appears to have been transferred to the national level including to the regional and local level of marine protected areas (MPAs).

Our pilot comparison of mentioning of genetic versus species diversity in Baltic Sea MPA management plans shows that species diversity is frequently occurring—all examined 34 plans mention this diversity several times but only 10 of the plans mention genetic diversity. Genetic search words constitute only around 1 percent of the total number of hits (2714 hits for species diversity and 30 hits for genetic diversity). This finding supports the notion that implementation of conservation policy for genetic biodiversity lags behind.

The lack of explicit genetic goals for MPAs in the Baltic Sea is unfortunate considering the particular importance of genetic diversity in this area (Johannesson et al. 2011); increasingly accumulating research indicates that genetic adaption to the particular environment has evolved and reflect ongoing speciation (Lamichhaney et al. 2012; Berg et al. 2015). Similarly, the importance of including genetic considerations in MPA management is increasingly highlighted (Arizmendi-Mejía et al. 2015; van der Meer et al. 2015).

We found that there is considerable complexity in the management structure of MPAs in the Baltic Sea. Several types of protection overlap each other partly or fully, plans are missing for 30 percent of HELCOM MPAs, and 12 percent of the areas are only partially covered by plans. Further, management plans are not easily accessible and are usually not available in English. This situation needs further attention in order to improve the potential for Baltic MPAs to actually protect biological diversity including the genetic level.

## Conclusion

International and national agreed policy on genetic biodiversity is not reflected in management plans for marine protected areas of the Baltic Sea. Management plans in all four countries that we investigated (Sweden, Finland, Estonia, Germany) are largely void of goals, concerns, strategies, or other mentioning of genetic biodiversity. This is in spite of area protection being expressed as an explicit measure to conserve genetic biodiversity in both international policy and in national implementation documents of these four countries. Thus, outspoken international goals of MPAs to function to conserve genetic diversity and to support gene flow among species appear not to be implemented at the regional level.

We suggest that one reason for this situation is that goals for genetic biodiversity are much less frequent in international and national policies directed toward aquatic environments (the Helsinki Convention, the EU Marine Strategy and Water Framework Directives) as compared to documents with a broader focus (CBD and the EU Habitats Directive). Other factors most likely also affect the situation and a better understanding of why implementing conservation genetic principles lags behind in marine environments is needed. Such factors could include lack of resources among regional policymakers and managers. We are addressing those issues for the Baltic Sea area in forthcoming studies that include interviews with managers and knowledge communication studies (Sandström et al., unpubl.; Lundmark et al., unpubl.). Several good examples of explicit goals and strategies for genetic conservation can be found in a few of the management plans for HELCOM MPAs (Tables 3, S1). It is important that these examples are spread and discussed among managers involved with MPA design and planning. Also, finding ways to bridge current gaps between conservation genetics researchers, policy makers, and managers (cf. Laikre et al. 2009) is necessary to achieve adaptive management of Baltic Sea genetic biodiversity.

## Electronic supplementary material

Below is the link to the electronic supplementary material.

Supplementary material 1 (PDF 489 kb)
