# Supplementary material for: Lack of recognition of genetic biodiversity: International policy and its implementation in Baltic Sea marine protected areas
Source: Ambio. 2016 Apr 20;45(6):661–80. doi: 10.1007/s13280-016-0776-7 (PMC5012997; doi:10.1007/s13280-016-0776-7)
Supplement: Supplementary file 1 — Supplementary material 1 (PDF 489 kb) [file 13280_2016_776_MOESM1_ESM.pdf]

Ambio

Electronic Supplementary Material
This supplementary material has not been peer reviewed.

Title: **Lack of recognition of genetic biodiversity: international policy and its implementation in Baltic Sea marine protected areas**

Authors: Linda Laikre, Carina Lundmark, Eeva Jansson, Lovisa Wennerström, Mari Edman, Annica Sandström

Appendix S1: **Baltic Sea Marine Protected Areas (MPAs) and our rational for focusing on HELCOM MPAs**

There are several types of MPAs in the Baltic Sea including variation among countries, legal status, etc. We initially intended to focus on two different types – Natura 2000 and HELCOM MPAs. Natura 2000 is a network of nature protection areas established under the EU Habitats Directive and/or the EU Birds Directive. Natura 2000 areas occur both on land and in water including in the Baltic Sea. Their governance are outlined and monitored by the EU Commission.

HELCOM MPAs (until recently called BSPAs, Baltic Sea Protected Areas) are coastal and marine protected areas in the Baltic Sea established under the Helsinki Commission (www.helcom.fi). They represent regional implementation of the Helsinki Convention. HELCOM MPAs are established based on HELCOM Recommendation 15/5 which was renewed in 2014 and is now superseded by the HELCOM Recommendation 35/1 (See Table 1).

Our rational for choosing these two types of MPAs (Natura 2000 and HELCOM MPAs) were that *i*) they represent types of marine protected areas that occur in all four countries that we chose to study, and *ii*) they represent different degree of legal commitment. The Natura 2000 network of conservation areas is linked to the implementation of the Habitat Directive (92/43/EEC) and the Birds Directive (2009/147/EEC) that are binding for all EU member states (including our four focal countries; Moussis 2015), whereas the HELCOM MPA system represents a non-binding agreement.

We identified HELCOM MPAs of the four separate focal countries from www.helcom.fi, and Natura 2000 that included at least some marine habitat using the Natura 2000 Network viewer (http://natura2000.eea.europa.eu/#), the Swedish Environmental Protection Agency´s map function (http://skyddadnatur.naturvardsverket.se/). We also contacted responsible authorities in the four countries to obtain lists of Natura 2000 with marine habitat in the Baltic Sea. To obtain management plans for the MPAs we searched the above sites, performed regular searches on the Internet, and contacted a range of authorities and contact persons of the separate countries.

During this work we became aware that *i*) HELCOM MPAs and Natura 2000 are, in most cases overlapping, *ii*) such overlap occur in all four countries that we investigated, although the degree of overlap varies both among and within countries, *iii*) specific management plans for HELCOM MPAs that are separate from the Natura 2000 plans rarely exist in any of the investigated countries, and *iv*) in all four countries the HELCOM MPAs and the Natura 2000 often overlap with other national forms of area protection. Further, we found that there are several hundred Natura 2000 sites in the Baltic Sea and that the management plans for them are not easily accessible in all countries. Also, it is not always straightforward to find out which areas include marine habitat and which areas only concern values on land. Thus, we chose to focus on HELCOM MPAs of the four countries and collected all available management plans that we were able to locate for protected areas within these MPAs.

Management complexity

There are a total of 64 HELCOM MPAs in the Baltic Sea countries we investigated; 20 Swedish ones, 33 Finnish, 7 Estonian, and 4 German (Fig. 3, Tables 3; S3). Finding management plans was not straightforward in any of the countries. We had to use Internet

searches, e-mail correspondence, as well as many telephone contacts before we were able to locate a total of 161 management plans that apply to 45 of the 64 HELCOM MPAs; 19 HELCOM MPAs lack management plans (1 in Sweden, 2 in Germany, and 16 in Finland, 11 out of which were established during 2014-2015).

In all four countries the HELCOM MPAs overlap with other types of protection including Natura 2000, and national protection measures such as national parks or nature reserves. Overall, the HELCOM MPAs include other types of protected areas with 1-35 such areas (average=3) per HELCOM MPA. Management responsibility varies among countries and rests with regional, county administrative boards (Sweden), regional authorities (Finland), federal states (Germany), and the national environmental board (Estonia).

The management plans have typically not been developed for the HELCOM MPA but for other types of protection that apply to the whole or parts of the same area (Natura 2000, national parks, or nature reserves). For 8 of the 45 HELCOM MPAs that have management plans the plans only cover part of the HELCOM MPA area (4 Estonian, 4 Swedish; Tables 3, S3).

In Sweden, each of the 20 HELCOM MPAs includes one or several other types of protection (Natura 2000, nature reserves, and/or national parks), and available management plans typically refer to those areas. Only in four cases single, overall plans for the entire HELCOM MPA have been developed (Table S3). Often the protected areas overlap in the sense that a Natura 2000 MPA also is a nature reserve or a national park. In such cases plans typically exist for both types of protection, yet referring to the same area. This management structure implies that there are a multitude of different management plans; we located a total of 132 management plans for the 20 Swedish HELCOM MPAs with between 1 and 35 plans per HELCOM MPA.

The situation is similar in Estonia, although at the most 7 protected areas are included in one HELCOM MPA. In one case, three types of protection apply to the same area: HELCOM MPA, Natura 2000, and national park regulation. All Estonian HELCOM MPAs have one or more management plans (Tables 3, S3).

In Finland there is usually a complete overlap between the various forms of protection such that HELCOM MPAs typically overlaps completely with a Natura 2000. Further, there can be additional overlap with national parks or other types of area protection in Finland. In many cases there is a complete overlap between a HELCOM MPA, a Natura 2000, and a national park such that the one and same area is protected in all three ways. Only one plan applies to a particular HELCOM MPA, however, and all the forms of protection are included in the same plan. In two cases one plan even covers several HELCOM MPAs including all other protected areas within them and in one case the plan refers to an even larger protected area which is not in itself a HELCOM MPA (Tables 3, S3).

There are no separate management plans for the German HELCOM MPAs. For the two areas where plans are available they apply to the national park located – in these two cases there is a complete overlap between HELCOM MPA, national park and Natura 2000 (Table S1).

Reference

Moussis N. 2015. Access to the European Union: law, economics, policies. 19th updated edition, Rixenstart, 2011. ISBN 978-2-9601045-0-9. Available at: http://www.europedia.moussis.eu

Table S1. Baltic Sea HELCOM MPAs in Sweden (20), Finland (33), Estonia (7) and Germany (4) included in this study (cf. Figure 3). In all four countries the HELCOM MPAs overlap with Natura 2000 and/or with other forms of protection such as national parks or nature reserves. Thus, in many cases a separate HELCOM MPA includes one or more Natura 2000, and in some cases it also includes a national park. The system for management plans varies among countries; in Finland if a management plan is available it covers the whole HELCOM MPA and all the other protected areas within it. Typically, there is complete overlap between HELCOM MPAs and Natura 2000 in Finland, and sometimes these also overlap completely with national parks. In contrast, in Sweden, Estonia, and Germany, when plans are available they apply to separate Natura 2000 or other protected areas within the MPA. In very few cases there are management plans that apply directly to the whole HELCOM MPA in these countries. No HMPAs=number of HELCOM MPAs, N2K=Natura 2000. No hits=number of times search terms (cf. Figure 2) occur in management plan(s), and the last column summarize if and how the hits refer to genetic diversity. *=the same one management plan refers to these three HELCOM MPAs. **one plan for two HELCOM MPAs

**Country (no HMPAs/ no plans)**	**HELCOM MPA**	**Fig 3 no**	**Natura 2000s in the MPA**	**Other protected areas in the MPA with management plans**	**Comments area overlaps**	**Available management plans**	**Plan(s) cover the HMPA area**	**Authority in charge**	**No hits/no plans with hits**	**Content on genetic diversity**
Finland (33/14)	Hailuoto northshore	22	Hailuoto, northshore FI1100201	-	Complete overlap HMPA – N2K.	1* for N2K: FI1100201	Yes	Metsähallitus	0/0	-*
	Isomatala-Maasyvänlahti	25	Isomatala–Maasyvänlahti FI1100203	-	Complete overlap HMPA – N2K.	1* for N2K: FI1100203	Yes	Metsähallitus	*	See results for Hailuoto.
	Eastern Gulf of Finland Archipelago and waters	53	Eastern Gulf of Finland Archipelago and waters FI0408001, Pernajabay and Pernaja Archipelago MPAs FI0100078	Eastern Gulf of Finland Archipelago National Park. Within this area is also another HELCOM MPA and an N2K: Pernajabay and Pernaja Archipelago MPAs (see below).	Within the area Eastern Gulf of Finland Archipelago and Waters there are several other protected areas. We think that it is	1 for N2K: FI0408001	Yes	Metsähallitus	0/0	-

					the National Park which provides the outer boarders for the whole area.					
	Kirkkonummi Archipelago	46	Kirkkonummi Archipelago FI0100026	-	Complete overlap HMPA – N2K.	No management plan	-	Metsähallitus	-	-
	Kirkkosalmi	26	KirkkosalmiFI 1100202	-	Complete overlap HMPA – N2K.	1* FI1100202	Yes	Metsähallitus	*	See results for Hailuoto.
	Kokkola Archipelago	29	Kokkola Archipelago FI1000033	-	Complete overlap HMPA – N2K.	1 FI1000033	Yes	Centre for Economic Develop-ment, Transport and the Environment (ELY Centre)	0/0	-
	Kristiinan-kaupunki Archipelago	33	Kristiinankaup unki Archipelago FI0800134	-	Complete overlap HMPA – N2K.	No management plan	-	Metsähallitus	-	-
	Liminka Bay	23	Liminka Bay FI1102200	-	Complete overlap HMPA – N2K.	No management plan	-	Metsähallitus	-	-
	Luoto Archipelago	28	Luoto Archipelago FI0800132	-	Complete overlap HMPA – N2K.	1 FI0800132	Yes	ELY Centre	0/0	-
	Outer Bothnian	31	Outer Bothnian	-	Complete	1 (draft)	Yes	Metsähallitus	0/0	-

	Threshold Archipelago - The Quark		Threshold Archipelago, the Quark FI0800130		overlap HMPA – N2K.	FI0800130				
	Närpiö Archipelago	32	Närpiö Archipelago FI0800135	-	Complete overlap HMPA – N2K.	No management plan	-	Metsähallitus	-	-
	Oura Archipelago	34	Oura Archipelago, FI0200077	Oura Archipelago national park. Complete overlap between HELCOM MPA, N2K, and the national park. Further, all these three areas are part of the larger Bothnian Sea National Park.	Complete overlap HMPA – N2K – National Park.<br><br>All three are part of another national park – Bothnian Sea National Park which is not in itself a HMPA.	1 (draft)** The plan for Oura Archipelago is included in the drafted management plan for the Bothnian Sea National Park.	Yes	Metsähallitus	1/1	Concerns mouflon sheep stating that their population size on an island should be large enough to maintain genetic diversity.
	Pernajabay and Pernaja Archipelago MPAs	50	Pernajabay and Pernaja Archipelago MPAs FI0100078	-	Complete overlap HMPA – N2K.	1 FI0100078 The new plan for Pernajabay and Pernaja Archipelago is included in the management plan for the Eastern Gulf of Finland Archipelago and waters.	Yes	Metsähallitus	0/0	-

	Bothnian Bay National Park	21	Bothnian Bay National Park FI1300301	Bothnian Bay National Park	Complete overlap HMPA – N2K.	1 (draft) FI1300301	Yes	Metsähallitus	0/0	-
	Porvoonjoki Estuary-Stensböle	49	Porvoonjoki Estuary-Stensböle FI0100074	-	Complete overlap HMPA – N2K.	1 FI0100074	Yes	ELY Centre	0/0	-
	Rahja Archipelago	27	Rahja Archipelago FI1000005	-	Complete overlap HMPA – N2K.	1 FI1000005 Included in a management plan for a larger protected area called Kalajoki Coast.	Yes	Metsähallitus	0/0	-
	Saaristomeri - Archipelago Sea	42	Saaristomeri - Archipelago Sea FI0200164	Saaristomeri National Park	Complete overlap HMPA, N2K, and national park.	1 For the National Park.	Yes	Metsähallitus	3/1	With respect to hunting important that genetic diversity is maintained. Gene banks potential aid for rare plants and animals.
	Söderskär and Långören Archipelago	47	Söderskär and Långören Archipelago FI0100077	-	Complete overlap HMPA – N2K.	1 (draft) FI0100077	Yes	Metsähallitus	0/0	-
	Tammisaari and	45	Tammisaari	Tammisaari and Hanko	The national	1	Yes	Metsähallitus	0/0	-

	Hanko Archipelago and Pojo Bay MPA		and Hanko Archipelago and Pojo Bay MPA FI0100005	Archipelago and Pojo Bay National Park	park is the largest of the three areas. Other areas included.	FI0100005				
	Tulliniemi bird protection area	43	Tulliniemi bird protection area FI0100006	-	Complete overlap HMPA – N2K.	1 (draft) FI0100006	Yes	Metsähallitus	0/0	
	Uusikaarlepyy Archipelago	30	Uusikaarlepyy Archipelago FI0800133	-	Complete overlap HMPA – N2K.	No management plan	-	Metsähallitus	-	-
	Uusikaupunki Archipelago	35	Uusikaupunki Archipelago FI0200072	Uusikaupunki Archipelago is part of the larger Bothnian Sea National Park	Complete overlap HMPA – N2K. Both part of larger national park which is not in itself a HMPA.	1(draft)** FI0200072 This plan is included in the draft management plan for the Bothnian Sea National park	-	Metsähallitus	**	See results for Oura Archipelago.
	Länsiletto	52	Länsiletto FI0400001	-	Complete overlap HMPA – N2K.	No management plan	-	-	-	-
	Luodematalat	51	Luodematalat FI0400002	-	Complete overlap HMPA – N2K.	No management plan	-	-	-	-
	Merikalla	24	Merikalla FI1100207	-	Complete overlap HMPA – N2K.	No management plan	-	-	-	-

	Björkör Islands	39	Björkör Islands FI1400006	-	Complete overlap HMPA – N2K.	No management plan	-	-	-	-
	Boxö Islands	36	Boxö Islands FI1400021	-	Complete overlap HMPA – N2K.	No management plan	-	-	-	-
	Långör-Östra Sundskär Islands	38	Långör-Östra Sundskär Islands FI1400042	-	Complete overlap HMPA – N2K.	No management plan	-	-	-	-
	Signilskär-Märket Islands	37	Signilskär-Märket Islands FI1400047	-	Complete overlap HMPA – N2K.	No management plan	-	-	-	-
	Sea area south from Sandkallan	48	Sea area south from Sandkallan FI0100106	-	Complete overlap HMPA – N2K.	No management plan	-	-	-	-
	Open sea area southeast from Hanko	44	Open sea area southeast from Hanko FI0100107	-	Complete overlap HMPA – N2K.	No management plan	-	-	-	-
	Lågskär Islands	40	Lågskär Islands FI1400058	-	Complete overlap HMPA – N2K.	No management plan	-	-	-	-
	Bogskär Islands	41	-	.	Only HMPA.	No management plan	-	-	-	-
Germany (4/2)	Kadetrinne	64	Kadetrinne DE1339301	-	Complete overlap HMPA – N2K.	No management plan	-	Bundesamt für Naturschutz, Insel Vilm	-	-

	Pommersche Bucht-Rönnebank	61	Westliche Rönnebank DE1249301, Adlergrund DE1251301, SPA Pommersche Bucht DE1552401, Pommersche Bucht mit Oderbank DE1652301	-		No management plan	-	Bundesamt für Naturschutz, Insel Vilm	-	-
	Jasmund National Park	62	Jasmund DE1447302	Jasmund National Park. Complete overlap between the HMPA, the N2K and the national park.	Complete overlap between the HMPA, the N2K and the national park.	1<br>For the national park.	Yes	Landesamt für umwelt, naturschutz und geologie	2/1	Aim of area includes preserving genetic resources of species.
	Vorpommersche Boddenlandschaft National Park	63	Recknitz-Ästuar und Halbinsel Zingst DE1542302, Darßer Schwelle DE1540302, Darß DE1541301, Vorpommersche Boddenlandschaft und nördlicher Strelasund	Vorpommersche Boddenland-schaft National Park. Complete overlap between HMPA and national park.	Complete overlap between HMPA and national park. 5 N2K included in this area.	1<br>One plan with three parts for the national park.	Yes	Landesamt für umwelt, naturschutz und geologie	3/1	Aim of area includes preserving genetic resources of species (2 hits). Baltic marine fish differ from North Sea populations. Two types of herring within the Baltic - spring and autumn spawning.

			DE1542401, Plantagenetgrund DE1343301							
Estonia (7/13)	Hiiu Madala	56	Paope EE0040112, Hiiu Madala EE0040129, Kõrgessaare-Mudaste EE0040130	-?		1 EE0040112	No	Estonian Environmental Board	0/0	-
	Kura Kurk	60	Kaugatoma-Lõu EE0040441, Abruka EE0040401, Allirahu EE0040402, Kura Kurgu EE0040434, Vesitükimaa EE0040490, Kerju EE0040421	Rahuste Nature Reserve		4 EE0040441, EE0040401, EE0040402, & Rahuste Nature Reserve	No	Estonian Environmental Board	0/0	-
	Lahemaa	54	Lahemaa EE0010173	Lahemaa National Park	Complete overlap between three types of protection.	1 EE0010173	Yes	Estonian Environmental Board	0/0	-
	Pakri	55	Pakri EE0010129			1 EE0010129	Yes	Estonian Environmental Board	0/0	-
	Pärnu lahe	58	Manilaiu-Hanilaiu			1 EE0040328	No	Estonian Environmen-	0/0	-

			EE0040328, Pärnu lahe EE0040346, Luitemaa EE0040351, Tõstamaa EE0040363					tal Board		
	Väinameri	57	Väikese väina EE0040486, Väinamere EE0040001	Hiiumaa Nature Reserve, Üügu Nature Reserve		3 EE0040486, Hiiuma Nature Reserve, & Üügu Nature Reserve	No	Estonian Environmen-tal Board	1/1	Assisted gene flow via pasturing cattle needed for disconnected marsh angelica (*Angelica palustris)* populations along seashores to avoid genetical impoverishment of the plant.
	Vilsandi	59	Tagamõisa EE0040476, Vilsandi EE0040496			2 EE0040476, EE0040496	Yes	Estonian Environmen-tal Board	1/1	Inbreeding a factor potentially affecting *Epidalea calamita* viability.
Sweden (20/129)	Hoburgs bank	5	Hoburgs bank SE0340144		Complete overlap HMPA and N2K.	1 SE0340144	Yes	Gotland County Administra-tive Board	0/0	-
	Kopparstenarna/ Gotska Sandön/Salvore v Area	7	Gotska sandön SE0340097	Gotska sandön National Park		2 SE0340097 & Gotska sandön National Park	Yes	Gotland County Administra-tive Board	1/1	Areas with no fishing allowed protect genetically valuable individuals of fish.
	Axmar	13	Axmar-Gåsholma SE0630166	Axmar Nature Reserve		2 SE0630166 & Nature Reserve	No	Gävleborg County Administra-tive Board	0/0	-

	Finngrundet-östra banken	14	Finngrundet-östra banken SE0630260		Complete overlap HMPA and N2K.	1 SE0630260	Yes	Gävleborg County Administra-tive Board	0/0	-
	Northern Midsjöbanken	4	Northern Midsjöbanken SE0330273		Complete overlap between HMPA & N2K.	No management plan		Kalmar County Administra-tive Board	-	
	Värnanäs Archipelago	3	Värnanäs Archipelago SE0330123	Värnanäs Archipelago Nature Reserve		2 SE0330123 & Nature Reserve	Yes	Kalmar County Administra-tive Board	1/1	Baltic harbor seal (*Phoca vitulina*) population genetically vulnerable due to small population size. Genetically separate from Swedish west coast populations.
	Haparanda Archipelago	20	Austi SE0820741, Haparanda skärgård SE0820108, Enskär SE0820742, Haparanda Sandskär SE0820320, Huitori SE0820743, Kataja SE0820744, Malören SE0820724,	Haparanda Archipelago National Park,<br>Malören Nature Reserve		15 SE0820741, SE0820108, SE0820742, SE0820320, SE0820743, SE0820744, SE0820724, SE0820734, SE0820746, SE0820735, SE0820747, SE0820748, SE0820750, the National Park & the	No	Norrbotten County Administra-tive Board	14/13	Risk of negative genetic effects due to isolated populations of Siberian primerose (*Primula nutans;* 13 plans) and bluntleaf sandwort (*Moehringia lateriflora;* 1 plan). The exact same text is repeated in 13 plans.

			Sarvenkataja SE0820734, Stora Hamnskär SE0820746, Stora Hepokari SE0820735, Tantamanni SE0820747, Tervaletto SE0820748, Äimä SE0820750			Nature Reserve				
	Marakallen	19	Marakallen SE0820751		Complete overlap HMPA and N2K.	1 SE0820751	Yes	Norrbotten County Administra-tive Board	0/0	-

	Falsterbo Peninsula with Måkläppen	1	Falsterbo skjutfält SE0430111, Falsterbo-Foteviken SE0430002, Falsterbohalvön SE0430095, Tygelsjö-Gessie SE043149, Vellinge ängar SE0430150	Flommen, Norra Ljungshusen, Skanör-Höll, Foteviksområdet, Skanörs Ljung, Kämpinge strandbad, and Ljungskogens och Ljungshusens strandbad Nature Reserves		12 One for each of the 5 N2K and one for each of the 7 nature reserves	No	Skåne County Administrative Board	4/1	Risk of negative genetic effects due to small population size for harbor seal *Phoca vitulina*, and due to small and isolated populations of little grape fern (*Botrychium simplex*; in plan for SE0430002).
	Torhamns Archipelago (Blekinge arkipelag)	2	Hästholmen-Öppenskär SE0410099, Sandhamn SE0410225, Torhamn-Hästholmen SE0410041, Torshamns udde SE0410104, Utlängan SE0410224	Torshamns Archipelago Helcom MPA, Hästholmen-Ytterön, Torhamns udde, Utklippan Nature Reserves	This HMPA has a particular plan for the whole area. There is overlap with nature reserves and N2K.	9 One for each N2K (5), one for each nature reserve (3), and one for the HMPA.	Yes	Blekinge County Administrative Board	0/0	-
	Bullerö-Bytta	10	Bullerö-Bytta SE0110088	Biskopsö, Bullerö, Långskär, and Långviksskär Nature	The five protected areas together	5 One for the N2K, one for	Yes	Stockholm County Administra-	0/0	-

				Reserves	cover the whole HMPA.	each nature reserve.		tive Board		
	Stora Nassa-Svenska Högarna	11	Stora Nassa SE0110092, Svenska Högarna SE0110096	Stora Nassa and Svenska Högarna Nature Reserves	This HMPA has a particular plan for the whole area.	5<br>One for each N2K, 2 for the nature reserves and one for the whole HMPA.	Yes	Stockholm County Administra-tive Board	8/3	Genetic exchange among populations needed for long term favourable status. Unclear if this goal is reached in this area. Risk of negative genetic effects due to small, isolated/poorly connected subpopulations of little grapefern (*Botrychium simplex*) and northern crested newt (*Triturus cristatus*; Stora Nassa SE0110092 and Svenska Högarna SE0110096). Restoring pike (*Esox lucius*) populations through releases should be documented and monitored to avoid negative genetic effects and inbreeding (Stora

										Nassa nature reserve).
	Fifång (Askö-Hartsö)	9	Fifång SE0110101, Skärtårdsreservaten Ringsö, Sävö, Lacka, Långö, Hartsö SE0220129, Bokö-Oxnö SE0220215, Stendörren SE0220218, Persö SE0220234, Askö SE0220439, Kråmö SE0220509	Fifång, Stora Bergö, Bokö-Askö, Bokö-Oxnö, Kråmö, Lacka, Långö, Persö, Askö, Stendörren, Sävö, Hartsö Nature Reserve	Several of the Nature Reserves overlaps completely with N2K. There are 5 plans for N2K SE0220129.	23<br>5 plans for the N2K SE0220129, one for each of the others (6). One for each nature reserve (12).	Yes	Stockholm and Södermanland County Administrative Boards	3/3	Fragmentation results in lack of gene flow between populations. An endemic subspecies of *Cakile maritima ssp. baltica* in Askö (SE0220439,SE0220129, Askö Nature Reserve).
	Gräsö-Singö Archipelago	12	Grillskäret SE0210248, Hållet, Blåbådan SE0210041, Högbådan SE0210249, Örskär SE0210228, Västerbådan, Lågagrundet SE0210040	Gräsö östra skärgård and Örskär Nature Reserves	Overlap between one nature reserve and a N2K (Örskär).	7<br>One for each N2K and each nature reserve.	No	Uppland County Administra-tive Board	10/3	Pool frog (*Rana lessonae*) which represents the genetically distinct Swedish population (Örskär) important to conserve (Örskär nature reserve and N2K). Genetically distinct populations often occur at distribution edges. A special section on genetic diversity and resiliens with several examples in

										plan for Gräsö östra skärgård nature reserve.
	Kronören	17	Kronören SE0810001	Kronören Nature Reserve	Complete overlap between HMPA, N2K, and nature reserve.	2<br>One each for N2K and nature reserve.	Yes	Västerbotten County Administra-tive Board	1/1	Risk of negative genetic effects due to isolation in the endangered *Hippuris tetraphylla*.
	The Holmö Islands	18	Holmöarna SE0810010	Holmöarna Nature Reserve	Complete overlap between HMPA, N2K, and nature reserve.	2<br>One each for N2K and nature reserve.	Yes	Västerbotten County Administra-tive Board	0/0	
	High Coast	16	Balesudden SE0710057, Djupviksberget SE0710181, Gnäggen SE0710143, Halsviksravinen SE0710046, Högbonden SE0710042, Hummelvik SE0710190, Lidängen SE0710216, Omneberget SE0710048, Ravin på Ronön SE0710204, Skuleskogen	Balesudden, Bråtan, Gnäggen, Herrestaberget, Hummelvik, Högbonden, Högklinten, Mjältön, Norrfällsviken, Omneberget, Rotsidan, Skuleberget, Trysunda, Halsviksravinen, Stormyran på Ulvön, Storsand, Storön, Södra Ulvön, Villmyran, Ögeltjärn Nature Reserves<br><br>Skuleskogens National Park<br><br>High coast Helcom MPA	This HMPA has a plan for the whole area.	35<br>One for each N2K (13), one for each nature reserve (20), one for the national park and one for the whole HMPA.	Yes	Västernorr-land County Administra-tive Board	14/2	Release of alien species, populations, or genes can cause genetic contamination and result in loss of genetic variation, genetic homogenization. Subspecies of conservation concern listed (Helcom MPA, Skuleskogen SE0710054).

			SE0710054, Stormyran på Ulvön SE0710182, Trysunda SE0710056, Villmyran SE0710142,							
	Vänta litets grund	15	Vänta litets grund SE0710225		Complete overlap between HMPA and N2K.	1 for N2K.	Yes	Västernorr-land County Administra-tive Board	0/0	
	Kvädöfjärden med Torrö	6	Ålsvikelandet-Kvädö SE 0230138	Kvädöfjärden and Torrö Nature Reserves		3 For N2K (1), and for the nature reserves (2).	Yes	Östergötland County Administra-tive Board	1/1	Protected areas important as gene banks to protect genetically distinct fish populations. For fishes protected areas conserve individuals that carries genes for rapid growth.
	S:t Anna-Missjö Archipelago	8	Sankt Anna och Gryts skärgårdar SE0230055, Missjö SE0230328	Missjö Nature Reserve<br><br>S:t Anna-Missjö Helcom MPA	This HMPA has a plan for the whole area.	4 One for the Helcom MPA, one each for the N2K and the nature reserve.	Yes	Östergötland County Administra-tive Board	4/2	Fragmentation of habitats results in lack of gene flow between populations (SE0230055). Eco-system approach to be applied aimed at securing all components of ecosystems including genetic

										variation. Probably genetic adaptation developed by Baltic Sea species. The population concept defined as a genetically separate group of individuals (HELCOM MPA plan).
